# Data-Driven Enhancements for MPC-Based Path Tracking Controller in Autonomous Vehicles

**DOI:** 10.3390/s24237657

**Published:** 2024-11-29

**Authors:** Jianhua Guo, Zhihao Xie, Ming Liu, Jincheng Hu, Zhiyuan Dai, Jinqiu Guo

**Affiliations:** 1 National Key Laboratory of Automotive Chassis Integration and Bionics, Changchun 130022, China; jiezh22@mails.jlu.edu.cn (Z.X.); daizy22@mails.jlu.edu.cn (Z.D.); jqguo23@mails.jlu.edu.cn (J.G.); 2 School of Automotive Studies, Tongii University, Shanghai 201804, China; 2110215@tongji.edu.cn; 3 Department of Aeronautical and Automotive Engineering, Loughborough University, Loughborough LE11 3TU, UK; j.hu2@lboro.ac.uk

**Keywords:** data-driven, model predictive control, path tracking

## Abstract

The accuracy of the control model is essential for the effectiveness of model-based control methods. However, factors such as model simplification, parameter variations, and environmental noise can introduce inaccuracies in vehicle state descriptions, thereby compromising the precision of path tracking. This study introduces data-driven enhancements for an MPC-based path tracking controller in autonomous vehicles (DD-PTC). The approach consists of two parts: firstly, Kolmogorov–Arnold Networks (KANs) are utilized to estimate tire lateral forces and correct tire cornering stiffness, thereby establishing a dynamic predictive model. Secondly, Gaussian Process Regression (GPR) is deployed to accurately capture the unmodeled dynamics of the vehicle to form a comprehensive control model. This enhanced model allows for precise path tracking through steering control. The superiority of DD-PTC is confirmed through extensive testing on the Simulink-CarSim simulation platform, where it consistently surpasses normal MPC and Linear Quadratic Regulator (LQR) strategies, especially in minimizing lateral distance errors under challenging driving conditions.

## 1. Introduction

Driven by rapid advancements in technology, autonomous vehicles have emerged as a significant focus of research, primarily due to their vast commercial potential and broad spectrum of applications. An autonomous driving system mainly consists of core modules such as perception, decision-making, planning, and tracking [1]. Among these, path tracking is especially critical as it directly influences the vehicle’s ability to precisely adhere to the planned path. Accurate path tracking is not only crucial for driving safety but also serves as an important indicator for evaluating the overall reliability of autonomous driving technologies [2]. Therefore, in-depth research and optimization of path tracking algorithms are crucial for enhancing the navigational accuracy and safety of autonomous vehicles. In recent years, a considerable amount of research has been conducted on path tracking technology for autonomous vehicles.

Currently, diverse algorithms are utilized in the design and optimization of path tracking controllers. These include proportional–integral–derivative control (PID), fuzzy logic control (FL), sliding mode control (SMC), linear quadratic regulator (LQR), robust control, and model predictive control (MPC) [3]. Compared to other controllers such as PID, LQR, and FL, MPC can more effectively handle constraints on state variables, control variables, and their rates of change, thereby achieving optimal control. Unlike SMC, MPC avoids potentially unsafe chattering phenomena, ensuring the smoothness of control actions [3].

In the design of path tracking controllers, the predictive model of MPC is a core component, essential for achieving high precision and stability in control. Kinematic single-track models, employed by researchers for path tracking applications [4,5,6,7], simplify the dynamics by assuming zero tire slip angles at both the front and rear tires and disregarding the effects of inertia. The primary benefits of this model are its minimal computational requirements and its effectiveness at lower speeds. However, the model’s accuracy diminishes with increased vehicle speed, limiting its effectiveness in high-speed scenarios. Dynamics single-track models, adopted by some researchers for path tracking [5,8,9,10], assume that the vehicle consistently maintains a constant speed and the tires remain within the linear region. Under these conditions, the lateral tire force maintains a linear relationship with the slip angle. At high speeds and with small steering angles, an MPC-based controller using the dynamics single-track model generally maintains good path tracking accuracy. When the tires exhibit nonlinear characteristics, the linear assumption of cornering stiffness leads to increased model error, which in turn severely affects tracking accuracy. Kabzan et al. [11] developed a hybrid model by combining the single-track kinematic model and the single-track dynamic model using an adjustable scaling factor. This approach improved the model’s adaptability but still faced the previously mentioned challenges. To more accurately capture real vehicle dynamics and enhance model precision, several researchers [12,13,14,15] have turned to the dynamics double-track model for path tracking, recognizing it as an effective approach. This model accounts for the forces exerted by each tire and can precisely simulate the effects of lateral load transfer, along with the impact of suspension springs and dampers, significantly enhancing the accuracy of path tracking. However, the complexity of the dynamics double-track model may restrict its widespread adoption in practical applications.

The aforementioned predictive models do not account for model errors resulting from factors such as model simplification, parameter variations, and environmental noise. This leads to inaccuracies in describing vehicle states, thereby affecting the accuracy of tracking. Researchers have developed various strategies to improve the accuracy and adaptability of models beyond the linear region. Several approaches [16,17,18,19,20] directly estimate tire cornering stiffness or precisely utilize tire force observers to estimate lateral forces and effectively adjust tire cornering stiffness to accommodate the nonlinear characteristics of the tires, thus significantly enhancing model performance and ensuring the accuracy of path tracking. In several studies [21,22,23,24,25], learning-based MPC strategies were proposed. These strategies integrate a simplified mechanistic model with residual models that are learned through data-driven methods. Employing an MPC framework, it effectively minimizes path tracking errors and has been validated for its effectiveness and practicality under the conditions of high speed and high acceleration. These advanced methods highlight the critical need to account for both nonlinear characteristics and unmodeled dynamics in dynamics models. By accurately estimating tire cornering stiffness or implementing learning-based MPC, these studies significantly improve the adaptability and predictive accuracy of the models. Despite individual advancements in enhancing path tracking through tire cornering stiffness estimation and data-driven approaches, integrated strategies that combine these methods are still relatively underexplored in the current research.

**Figure 1:**
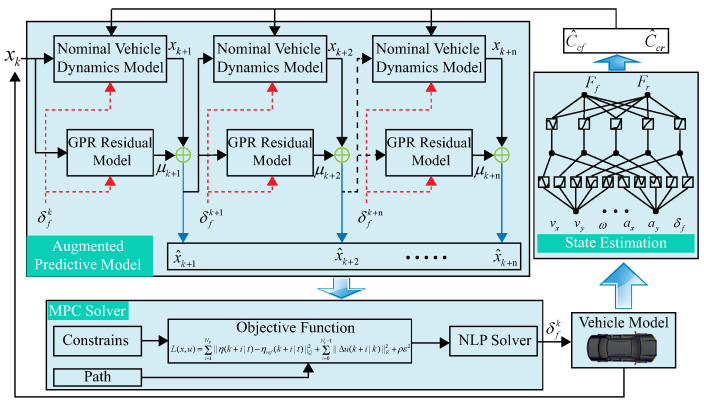
Framework of DD-PTC.

Inspired by the methods discussed above, this study introduces a data-driven enhancements for an MPC-based path tracking controller architecture that aims to improve path tracking accuracy through precise modeling of the predictive model. As shown in Figure 1, the architecture includes three main components: the state estimation layer, the augmented predictive model layer, and the MPC solver layer. Firstly, we utilize the KAN network [26] to estimate the lateral forces on the front and rear axles of the vehicle, which are employed to correct the vehicle dynamics model. Based on this, GPR is employed as the residual model to describe the deviation between the simplified mechanistic model and the actual vehicle state. Finally, the MPC-based controller is employed to solve for the optimal control solutions. The main contributions of this paper include the following:

- In response to the challenge that the relationship between the lateral force and slip angle becomes nonlinear as the tires exhibit nonlinear characteristics, this study employs the KAN network to estimate the lateral forces to correct the tire cornering stiffness, thereby enhancing the accuracy of the predictive model.
- GPR is utilized to learn the residual dynamics between the simplified mechanistic model and the actual vehicle state. This strategy significantly enhances the predictive model accuracy of the MPC and further improves the precision of path tracking.

The remainder of this paper is organized as follows: Section 2 introduces the establishment of the vehicle prediction model; Section 3 elaborates on the data-driven enhancements for the MPC-based path tracking controller; Section 4 presents the simulation results to demonstrate the effectiveness of the strategy; and Section 5 discusses the conclusions.

## 2. Nominal Vehicle Dynamics Model

**Figure 2:**
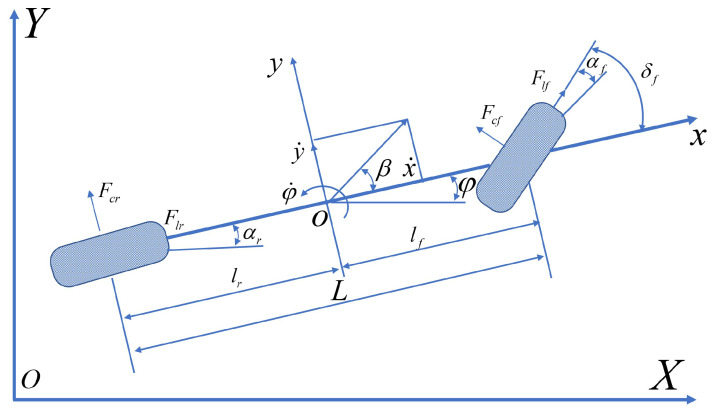
Dynamic model.

When autonomous vehicles follow a path, the primary emphasis is placed on assessing the vehicle’s lateral dynamics performance. The effects of air resistance and load transfer between the front and rear axles on tire characteristics are ignored. A three-degree-of-freedom vehicle dynamics model encompassing longitudinal, lateral, and yaw motion is established, as shown in Figure 2.

The vehicle dynamics equations are as follows:

(1) $$\left\{\begin{array}{c} m\ddot{x}=m\dot{y}\dot{\varphi }+2F_{xf}+2F_{xr}\\ m\ddot{y}=-m\dot{x}\dot{\varphi }+2F_{yf}+2F_{yr}\\ I_{z}\ddot{\varphi }=2l_{f}F_{yf}-2l_{r}F_{yr} \end{array}\right.$$

where *m* represents the mass of the vehicle, and $l_{f}$ and $l_{r}$ denote the distances from the vehicle’s center of mass to the front and rear axles, respectively. In the vehicle coordinate system, *x* and *y* represent the longitudinal and lateral positions, $\dot{x}$ and $\dot{y}$ represent the longitudinal and lateral speeds, and $\ddot{x}$ and $\ddot{y}$ represent the longitudinal and lateral accelerations. φ, $\dot{\varphi }$, and $\ddot{\varphi }$ correspond to the vehicle’s yaw angle, yaw rate, and yaw acceleration, respectively. $I_{z}$ denotes the vehicle’s moment of inertia. $F_{xf}$ and $F_{yf}$ represent the decomposition of the front tire forces into lateral and longitudinal components, while $F_{xr}$ and $F_{yr}$ represent the decomposition of the rear tire forces into lateral and longitudinal components, respectively, as follows:

$$\begin{array}{c} \left\{\begin{array}{c} F_{xf}=F_{lf}\cos\delta_{f}-F_{cf}\sin\delta_{f}\\ F_{xr}=F_{lr}\\ F_{yf}=F_{lf}\sin\delta_{f}+F_{cf}\cos\delta_{f}\\ F_{yr}=F_{cr} \end{array}\right. \end{array}$$

where $F_{lf}$ and $F_{lr}$ represent the longitudinal forces of the front and rear wheels, respectively, while $F_{cl}$ and $F_{cr}$ represent the lateral forces of the front and rear wheels, respectively. Considering the slip angle and slip rate are within a small range near the origin, the lateral and longitudinal forces of the tires can be simplified into a linear relationship, as follows:

(2) $$\left\{\begin{array}{c} F_{l}=C_{l}s\\ F_{cf}=C_{cf}\alpha_{f}=C_{cf}\left(\frac{l_{f}\dot{\varphi }+\dot{y}}{\dot{x}}-\delta_{f}\right)\\ F_{cr}=C_{cr}\alpha_{r}=C_{cr}\frac{\dot{y}-l_{r}\dot{\varphi }}{\dot{x}} \end{array}\right.$$

where $C_{l}$ represents the longitudinal stiffness of the tires, and $C_{c,i}$ with i∈{f,r} represents the cornering stiffness of the tires, respectively. $\alpha_{i}$ with i∈{f,r} represents the tire slip angle, μ denotes the ground adhesion coefficient, and *s* indicates the tire slip rate.

**Figure 3:**
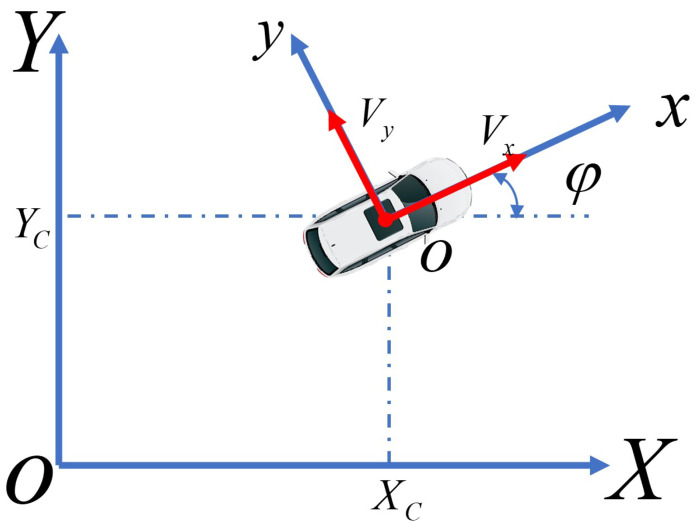
Coordinate transform.

Considering the transformation relationship between the coordinate system of the vehicle and the inertial coordinate system, as shown in Figure 3, the coordinate transformation formula can be expressed as follows:

(3) $$\left\{\begin{array}{c} \dot{Y}=\dot{x}\sin\varphi +\dot{y}\cos\varphi \\ \dot{X}=\dot{x}\cos\varphi -\dot{y}\sin\varphi \end{array}\right.$$

Finally, the single-track model of vehicle dynamics can be described as follows:

(4) $$\begin{array}{c} \dot{\xi }\left(t\right)=f_{dyn}\left(\xi \left(t\right),u\left(t\right)\right)=\left\{\begin{array}{c} \dot{x}\cos\varphi -\dot{y}\sin\varphi \\ \dot{x}\sin\varphi +\dot{y}\cos\varphi \\ \dot{\varphi }\\ \frac{1}{m}\dot{y}\dot{\varphi }+2\left(C_{lf}s_{f}+C_{lr}s_{r}+C_{cf}\alpha_{f}\delta_{f}\right)\\ \frac{1}{m}\left(-m\dot{x}\dot{\varphi }+2C_{cf}\alpha_{f}+2C_{cr}\alpha_{r}\right)\\ \frac{2}{I_{z}}\left(aC_{cf}\alpha_{f}-bC_{cr}\alpha_{r}\right) \end{array}\right. \end{array}$$

In Equation (4), the state variables and control variable are as follows:

(5) $$\left\{\begin{array}{c} \xi ={\left[\begin{array}{cccccc} X & Y & \dot{x} & \dot{y} & \varphi & \dot{\varphi } \end{array}\right]}^{T}\\ u=\delta_{f} \end{array}\right.$$

To meet computational requirements, the model described above must be linearized. The linearized equations can be rewritten as follows:

(6) $$\dot{\xi }=f\left(\xi (t),u(t)\right)$$

By performing a Taylor expansion around the reference point $\left(\xi_{t},u_{t}\right)$, Equation (6) can be approximately transformed into a linear time-varying (LTV) system.

(7) $$\dot{\xi }=A\xi +Bu$$

where

(8) $$A=\frac{\partial f}{\partial x}{|}_{\xi_{t},u_{t}}=\left[\begin{array}{cccccc} -\frac{2\left(C_{cf}+C_{cr}\right)}{m{\dot{x}}_{t}} & \frac{\partial f_{\dot{y}}}{\partial \dot{x}} & 0 & -{\dot{x}}_{t}+\frac{2\left(l_{r}C_{cr}-l_{f}C_{cf}\right)}{m{\dot{x}}_{t}} & 0 & 0\\ \dot{\varphi }-\frac{2C_{cf}\delta_{f,t-1}}{m{\dot{x}}_{t}} & \frac{\partial f_{\dot{x}}}{\partial \dot{x}} & 0 & {\dot{y}}_{t}-\frac{2l_{f}C_{cf}\delta_{f,t-1}}{m{\dot{x}}_{t}} & 0 & 0\\ 0 & 0 & 0 & 1 & 0 & 0\\ \frac{2\left(l_{r}C_{cr}-l_{f}C_{cf}\right)}{I_{z}{\dot{x}}_{t}} & \frac{\partial f_{\dot{\varphi }}}{\partial \dot{x}} & 0 & -\frac{2\left({l_{f}}^{2}C_{cf}+{l_{r}}^{2}C_{cr}\right)}{I_{z}{\dot{x}}_{t}} & 0 & 0\\ \cos\left(\varphi_{t}\right) & \sin\left(\varphi_{t}\right) & {\dot{x}}_{t}\cos\left(\varphi_{t}\right)-{\dot{y}}_{t}\sin\left(\varphi_{t}\right) & 0 & 0 & 0\\ -\sin\left(\varphi_{t}\right) & \cos\left(\varphi_{t}\right) & -{\dot{y}}_{t}\cos\left(\varphi_{t}\right)-{\dot{x}}_{t}\sin\left(\varphi_{t}\right) & 0 & 0 & 0 \end{array}\right]$$

(9) $$B=\frac{\partial f}{\partial u}{|}_{\xi_{t},u_{t}}=\left[\frac{2C_{cf}}{m},\frac{2C_{cf}\left(2\delta_{f,t-1}-\frac{{\dot{y}}_{t}+l_{f}{\dot{\varphi }}_{t}}{{\dot{x}}_{t}}\right)}{m},0,\frac{2l_{f}C_{cf}}{I_{z}},0,0\right]$$

(10) $$\frac{\partial f_{\dot{y}}}{\partial \dot{x}}=\left(2C_{cf}\left({\dot{y}}_{t}+l_{f}{\dot{\varphi }}_{t}\right)+2C_{cr}\left({\dot{y}}_{t}-l_{r}{\dot{\varphi }}_{t}\right)\right)/m{\dot{x}}_{t}^{2}-{\dot{\varphi }}_{t}$$

(11) $$\frac{\partial f_{\dot{\varphi }}}{\partial \dot{x}}=\left(2l_{f}C_{cf}\left({\dot{y}}_{t}+l_{f}{\dot{\varphi }}_{t}\right)-2l_{r}C_{cr}\left({\dot{y}}_{t}-l_{r}{\dot{\varphi }}_{t}\right)\right)/I_{z}{\dot{x}}_{t}^{2}$$

## 3. DD-PTC Architecture

### 3.1. Augmented Predictive Model Layer

#### 3.1.1. Gaussian Process Regression

Inspired by [21,22], we employ GPR to augment the nominal dynamics model in the MPC framework. A GPR ensemble predicts dynamics model errors and makes adjustments at each time step. Similar to most GP-based learning problems, we assume a true vehicle dynamics model, $f_{true}$. We obtain ${\tilde{y}}_{k+1}$ by measuring at discrete time points $t_{k}$, where the measurements are subject to noise.

(12) $${\tilde{y}}_{k+1}=f_{true}\left(x_{k},u_{k}\right)+w_{k}$$

Assuming Gaussian noise $w_{k}∼N\left(0,\Sigma \right)$ with a time-invariant diagonal covariance matrix, this implies that each output dimension can be independently processed using a one-dimensional GPR. GPR employs the Radial Basis Function (RBF) as its kernel function, defined as follows:

(13) $$\kappa \left(z_{i},z_{j}\right)=\sigma_{f}^{2}exp\left(-\frac{1}{2}{\left(z_{i}-z_{j}\right)}^{T}L^{-2}\left(z_{i}-z_{j}\right)\right)+\sigma_{n}^{2}$$

where *L* represents the diagonal length scale matrix, $\sigma_{f}$ and $\sigma_{n}$ denote the data variance and the prior noise variance, respectively, while $z_{i}$ and $z_{j}$ represent data features.

The vehicle system dynamics is redefined as a combination of the normal dynamics plus the mean posterior $\mu_{gp}$ from the GPR, and $\mu_{gp}$ is determined by the selection matrix $B_{d}$ and the input feature vector $z_{k}$.

(14) $$f_{cor}\left(x_{k},u_{k}\right)=f_{dyn}\left(x_{k},u_{k}\right)+B_{d}\mu_{gp}\left(z_{k}\right)$$

In this paper, the input to the GPR is selected as $z_{k}={\left[\begin{array}{ccc} \dot{y} & \varphi & \dot{\varphi } \end{array}\right]}^{T}$, with $B_{d}\mu \left(z_{k}\right)$ representing the residual compensation for the state variables. This paper focuses on correcting $\left[\begin{array}{cccc} \dot{y} & \varphi & \dot{\varphi } & Y \end{array}\right]$. Given the training feature samples *Z* and the query feature sample $z_{k}$, the mean and covariance predicted by GPR can be derived as follows:

(15) $$\mu_{gp}\left(z_{k}\right)=K_{k}^{T}K^{-1}Z$$

(16) $$\Sigma_{uk}=K_{kk}-K_{k}^{T}K^{-1}K_{k}$$

where $K,\;K_{k}$, and $K_{kk}$, respectively, represent:

(17) $$K=\kappa \left(Z,Z\right)+\sigma_{n}^{2}I$$

(18) $$K_{k}=\kappa \left(Z,Z_{k}\right)$$

(19) $$K_{kk}=\kappa \left(Z_{k},Z_{k}\right)$$

#### 3.1.2. Data Collection and Practical Implementation

To fit the GPR ensemble, data are collected in Carsim using the nominal dynamics model. For each sampling moment $t_{k}$, the actual state variables at the next time step $x_{k+1}^{true}$, the state variables predicted by the prediction model $x_{k+1}$, and the time step $\delta t_{k}$ are recorded. The error in the state variables of the vehicle per unit time is then calculated as follows:

(20) $$x_{ek}=\frac{x_{k+1}^{true}-x_{k+1}}{\delta t_{k}}$$

During the fitting process of GPR, outlier processing is initially conducted on the collected data. Subsequently, to reduce the fitting errors of GPR, we use a Gaussian Mixture Model (GMM) to cluster the data, and a separate GPR is fitted for each category to form a GPR ensemble. Better fitting results are achieved by appropriately selecting the hyperparameters of the kernel function. During this process, we utilize the maximum likelihood estimation method to optimize the hyperparameters of the kernel function, ensuring that the predictive model can accurately reflect the actual vehicle dynamics. In practical applications, by calculating the Euclidean distance between the current vehicle state and each cluster center, the GPR model from the closest category is chosen from the GPR ensemble to correct state variable errors in MPC. This method effectively improves prediction accuracy and optimizes control performance.

### 3.2. State Estimation Layer

The tire lateral force calculated based on constant cornering stiffness only provides good approximations under stable conditions. When there are drastic changes in the vehicle’s vertical load, significant discrepancies arise between the calculated tire lateral force using constant stiffness and the actual lateral force. Therefore, fixed stiffness values $C_{cf}$ and $C_{cr}$ struggle to adapt to scenarios with significant changes in vertical load. To enhance path tracking performance in autonomous vehicles, a lateral force observer based on the KAN network has been designed. This observer can precisely estimate the tire’s lateral force, and then the cornering stiffness is dynamically adjusted.

The KAN network is based on the Kolmogorov–Arnold representation theorem, which proves that any multivariate function can be represented by a sum of single-variable functions. Compared to Multilayer Perceptrons (MLPs) [27], KAN networks offer several advantages. Firstly, KAN networks have better interpretability and exhibit faster neural scaling laws than MLPs. By leveraging the locality of splines, KAN networks can avoid the catastrophic forgetting issue, which is a severe problem observed in MLPs. Secondly, KAN networks achieve comparable performance to MLPs with fewer parameters and exhibit good generalization capabilities [26].

Assuming the structure of the KAN is $\left[n_{0},n_{1},\cdots ,n_{L}\right]$, where $n_{i}$ represents the number of nodes in the $i^{th}$ layer of the computational graph, we denote $i^{th}$ the neuron in the $l^{th}$ layer as $\left(l,i\right)$, and its activation value as $x_{l,i}$. The activation function connecting $\left(l,i\right)$ and $\left(l,j+1\right)$ is denoted as follows:

(21) $$\phi_{l,j,i},l=0,\cdots ,L-1,i=1,\cdots ,n_{l},j=1,\cdots ,n_{l+1}.$$

Given the pre-activation value of $x_{l,i}$, the post-activation value ${\tilde{x}}_{l,j,i}$ is obtained through the activation function $\phi_{l,j,i}$. The activation value $\left(l+1,j\right)$ is the sum of all ${\tilde{x}}_{l,j,i}$.

(22) $$x_{l+1,j}=\sum_{i=1}^{n_{l}}{\tilde{x}}_{l,j,i}=\sum_{i=1}^{n_{l}}\phi_{l,j,i}\left(x_{l,i}\right),i=1,\cdots ,n_{l},j=1,\cdots ,n_{l+1}.$$

The matrix representation of the above Equation (22) is given by the following:

(23) $$x_{l+1}=\underset{\Phi_{l}}{\underset{︸}{\left(\begin{array}{cccc} \phi_{l,1,1}\left(\cdot \right) & \phi_{l,1,2}\left(\cdot \right) & \cdots & \phi_{l,1,n_{l}}\left(\cdot \right)\\ \phi_{l,2,1}\left(\cdot \right) & \phi_{l,2,2}\left(\cdot \right) & \cdots & \phi_{l,2,n_{l}}\left(\cdot \right)\\ \vdots & \vdots & & \vdots \\ \phi_{l,n_{l+1},1}\left(\cdot \right) & \phi_{l,n_{l+1},2}\left(\cdot \right) & \cdots & \phi_{l,n_{l+1},n_{l}}\left(\cdot \right) \end{array}\right)}}x_{l}$$

Given an input vector $x_{0}\in R^{n_{0}}$, the output of the KAN network is denoted by the following:

(24) $$KAN\left(x\right)=\left(\Phi_{L-1}\circ \Phi_{L-2}\circ \cdots \circ \Phi_{1}\circ \Phi_{0}\right)x$$

where $\Phi_{l}$ is the function matrix corresponding to the $l^{th}$ KAN layer. The selected input features for the KAN network are $z_{k}=\left[\begin{array}{cccccc} vx & vy & yaw\_rate & ax & ay & delta \end{array}\right]$, and the output of the KAN network is as follows:

(25) $$\left[{\hat{F}}_{cf},{\hat{F}}_{cr}\right]=KAN\left(z_{k}\right)$$

where ${\hat{F}}_{cf}$ and ${\hat{F}}_{cr}$ are the estimated lateral forces. The tire cornering stiffness is adjusted based on the lateral tire force estimated by the KAN network. The tire cornering stiffness correction coefficient is defined as follows:

(26) $$\left\{\begin{array}{c} \lambda_{f}=\frac{{\hat{F}}_{cf}-F_{cf}}{|{\hat{F}}_{cf}|}=\frac{{\hat{F}}_{cf}-C_{cf}\left(\frac{l_{f}\dot{\varphi }+\dot{y}}{\dot{x}}-\delta_{f}\right)}{{\hat{F}}_{cf}}\\ \lambda_{r}=\frac{{\hat{F}}_{cr}-F_{cr}}{|{\hat{F}}_{cr}|}=\frac{{\hat{F}}_{cr}-C_{cr}\left(\frac{\dot{y}-l_{r}\dot{\varphi }}{\dot{x}}\right)}{{\hat{F}}_{cr}} \end{array}\right.$$

The corrected tire cornering stiffness is given by the following equation:

(27) $$\left\{\begin{array}{c} {\hat{C}}_{cf}=\left(1+\lambda_{f}\right)C_{cf}\\ {\hat{C}}_{cr}=\left(1+\lambda_{r}\right)C_{cr} \end{array}\right.$$

To avoid the failure of tire lateral force estimation and the deterioration of the path tracking controller’s performance due to excessive correction by the correction factor, the correction coefficients for the cornering stiffness of the front and rear axles are constrained as follows:

(28) $$\left\{\begin{array}{c} \lambda_{f\;\min}\leq \lambda_{f}\leq \lambda_{f\;\max}\\ \lambda_{r\;\min}\leq \lambda_{r}\leq \lambda_{r\;\max} \end{array}\right.$$

where $\lambda_{f\;\max}$ and $\lambda_{f\;\min}$ are the upper and lower limits of the front axle cornering stiffness correction coefficient, while $\lambda_{r\;\max}$ and $\lambda_{r\;\min}$ are the upper and lower limits for the rear axle cornering stiffness correction coefficient. The lower and upper limits are set as $\lambda_{f\;\min}=\lambda_{r\;\min}=-0.4$ and $\lambda_{f\;\max}=\lambda_{r\;\max}=0.4$, respectively. The cornering stiffness in the path tracking controller is replaced with the corrected cornering stiffnesses ${\hat{C}}_{cf}$ and ${\hat{C}}_{cr}$. To prevent singularities in the correction factor when the tire lateral force is too low, the correction coefficient is set to zero if the tire slip angle’s absolute value is less than $0.1^{\circ }$.

### 3.3. MPC Solver Layer

MPC controls a system subject to its dynamics $\dot{x}=f\left(x,u\right)$ by minimizing a cost function $L\left(x,u\right)$ based on reference outputs $\eta_{ref}\left(t\right)$ and reference input controls $u^{*}\left(t\right)$. The general form of MPC is as follows:

(29) $$\begin{array}{c} \min_{u}\int L\left(x,u\right)\\ s.t.\dot{x}=f_{dyn}\left(x,u\right)\\ x_{0}=x_{init}\\ r(x,u)=0\\ h(x,u)\leq 0 \end{array}$$

where $x_{0}$ is the initial state, and *r* and *h* represent the equality and inequality constraints, respectively. $\dot{x}=f_{dyn}\left(x,u\right)$ can be further written in the form of state space equations $\dot{x}=A_{\mathrm{dis}}x+B_{\mathrm{dis}}u$, and the output is $\eta =C_{\mathrm{dis}}x+D_{\mathrm{dis}}u$. The normal model introduced in Section 2 is employed here. The cost function and state space equations above are expressed in a continuous form. For practical applications, it is necessary to discretize the equations. Assuming the discretization time step is *T*, the output in this study is solely dependent on *x*. The forward Euler method is used for discretization to obtain the following:

(30) $$\left\{\begin{array}{c} x\left(k+1\right)=A_{k}x\left(k\right)+B_{k}u\left(k\right)\\ \eta \left(k\right)=C_{k}x\left(k\right) \end{array}\right.$$

where $A_{k}=I+A_{\mathrm{dis}}T$, $B_{k}=B_{\mathrm{dis}}T$, $C_{k}=C_{\mathrm{dis}}$, and T=0.01. To prevent sudden changes in the control variables that could cause danger during the driving process, it is necessary to limit the control increments. Thus, the augmented state vector is defined as follows:

(31) $$\chi \left(k\right)={\left[\begin{array}{cc} \tilde{\xi }\left(k\right) & \tilde{u}\left(k-1\right) \end{array}\right]}^{T}$$

The new expression for the state space equations is obtained as follows:

(32) $$\chi \left(k+1\right)=A\chi \left(k\right)+B\Delta u\left(k\right)$$

(33) y(k)=Cχ(k)

where $A=\left[\begin{array}{cc} A_{k} & B_{k}\\ 0_{m\times n} & I_{m} \end{array}\right]$, $B=\left[\begin{array}{c} B_{k}\\ I_{m} \end{array}\right]$, $C=[\begin{array}{cc} C_{k} & 0_{m\times 1} \end{array}]$, *n* is the number of state variables, and *m* is the number of control variables. The state prediction incorporating GPR-augmented dynamics can be expressed as follows:

(34) $$\begin{array}{c} \chi \left(k+1|k\right)=A\chi \left(k\right)+B\Delta u\left(k|k\right)+\mu_{gp}\left(\xi \left(k\right)\right)\\ \chi \left(k+2|k\right)=A^{2}\chi \left(k\right)+AB\Delta u\left(k|k\right)+B\Delta u\left(k+1|k\right)+\mu_{gp}\left(\xi \left(k+1|k\right)\right)\\ \chi \left(k+3|k\right)=A^{3}\chi \left(k\right)+A^{2}B\Delta u\left(k|k\right)+AB\Delta u\left(k+1|k\right)+B\Delta u\left(k+2|k\right)+\mu_{gp}\left(\xi \left(k+2|k\right)\right)\\ \vdots \\ \chi \left(k+N_{p}|k\right)=A^{N_{p}}\chi \left(k\right)+A^{N_{p}-1}B\Delta u\left(k|k\right)+A^{N_{p}-2}B\Delta u\left(k+1|k\right)\\ +\cdots +A^{N_{p}-N_{c}}B\Delta u\left(k+N_{c}-1|k\right)+\mu_{gp}\left(\xi \left(k+N_{p}-1|k\right)\right) \end{array}$$

where $\mu_{gp}$ is the mean of the state variable errors predicted by GPR, $N_{p}$ is the prediction horizon of the system, $N_{c}$ is the control horizon, and Δu represents the control increments. The output of the system at future time steps is given by the following:

(35) $$\begin{array}{c} \eta (k+1|k)=C\chi (k+1|k)\\ \eta (k+2|k)=C\chi (k+2|k)\\ \eta (k+3|k)=C\chi (k+3|k)\\ \vdots \\ \eta \left(k+N_{p}|k\right)=C\chi \left(k+N_{p}|k\right) \end{array}$$

The primary goal of the cost function is to penalize deviations between the output and the reference target, and to minimize the rate of change in control actions as much as possible, thereby ensuring passenger comfort. Additionally, to prevent situations where no solution is feasible, a slack variable ε is introduced. In this study, we specify the cost function in a quadratic form.

(36) $$L\left(x,u\right)=\overset{N_{p}}{\underset{i=1}{\Sigma }}||\eta \left(k+i|t\right)-\eta_{ref}{\left(k+i|t\right)||}_{Q}^{2}+\overset{N_{c}-1}{\underset{i=0}{\Sigma }}{\left||\Delta u\left(k+i|k\right)|\right|}_{R}^{2}+\rho \varepsilon^{2}$$

The first summation term penalizes the difference between the expected vehicle state and the state within the prediction horizon of $N_{p}$ steps. The second summation term penalizes the control increments over the control horizon of $N_{c}$ steps. *Q* and *R* are the weight matrices. In addition, a set of constraints is applied to the control actions.

(37) $$u_{\min}\leq u\left(k\right)\leq u_{max}$$

The sequence of control actions and the sequence of control increments satisfy the following relationship:

(38) U(k)=MΔU(k)+Γu(k−1)

where $U\left(k\right)=\left[\begin{array}{c} u(k|k)\\ u(k+1|k)\\ \vdots \\ u(k+N_{c}-1|k) \end{array}\right]$, $M=\left[\begin{array}{cccc} I_{m} & 0 & \cdots & 0\\ I_{m} & I_{m} & \cdots & 0\\ \vdots & \vdots & \ddots & 0\\ I_{m} & I_{m} & I_{m} & I_{m} \end{array}\right]$, $\Gamma =\left[\begin{array}{c} I_{m}\\ I_{m}\\ \vdots \\ I_{m} \end{array}\right]$,

$\Delta U\left(k\right)=\left[\begin{array}{c} \Delta u(k|k)\\ \Delta u(k+1|k)\\ \vdots \\ \Delta u(k+N_{c}-1|k) \end{array}\right]$.

According to Equation (37), constraints are imposed on the sequence of control actions as follows:

(39) $$U_{\min}\leq U\left(k\right)\leq U_{\max}$$

The constraints for the front wheel steering angle and the increment of the front wheel steering angle are set as follows:

(40) $$\left\{\begin{array}{c} -30\leq u\leq 30\\ -0.47\leq \Delta u\leq 0.47 \end{array}\right.$$

In summary, the constraints can be represented by the following equation:

(41) $$\left[\begin{array}{c} M\\ -M\\ I\\ -I \end{array}\right]\Delta U\left(k\right)\leq \left[\begin{array}{c} U_{\max}-\Gamma u\left(k-1\right)\\ -U_{\min}+\Gamma u\left(k-1\right)\\ \Delta U_{\max}\\ -\Delta U_{\min} \end{array}\right]$$

Finally, the solution is obtained using a nonlinear programming (NLP) solver.

## 4. Simulation and Analysis

**Figure 4:**
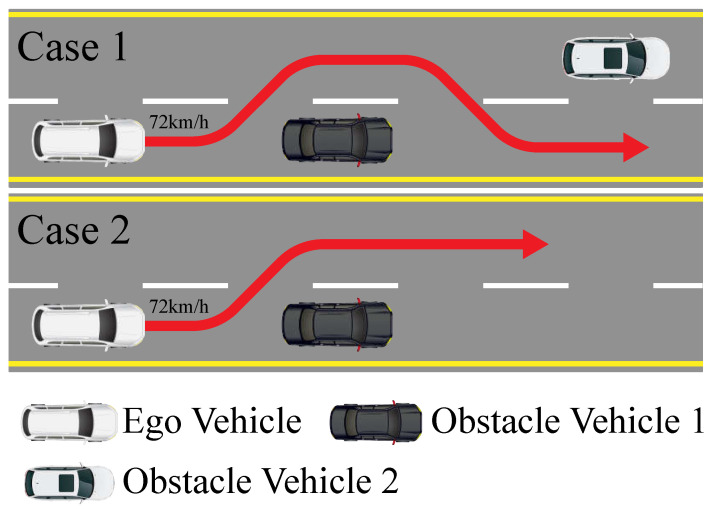
The traffic scenes of two cases.

To validate the effectiveness and robustness of the proposed method, we selected two typical scenarios for path tracking verification, as illustrated in Figure 4. These scenarios correspond to double-lane change and single-lane change maneuvers. Both scenarios were simulated using the Simulink-CarSim joint simulation platform. The parameters for the vehicle in the CarSim simulation model are detailed in Table 1. To demonstrate the superiority of DD-PTC, path tracking controllers based on MPC and LQR were developed as benchmarks.

### 4.1. Estimation Results of the KAN Model

**Figure 5:**
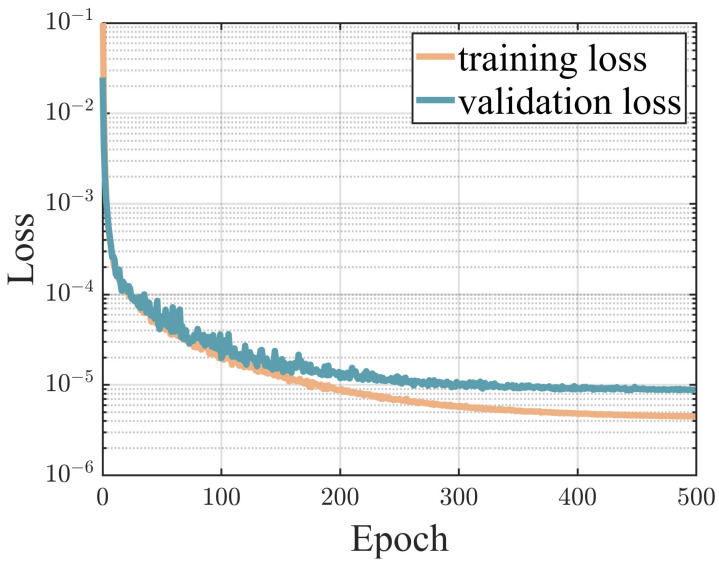
Learning curve.

As discussed in Section 3, the lateral force was estimated using the KAN network. Data were gathered via Carsim across a variety of driving scenarios and conditions. Subsequently, these data were employed to train the KAN network, which is structured as follows: the input layer consists of six neurons, corresponding to the primary variables influencing the outputs. This is followed by a hidden layer containing 32 neurons, which allows for the extraction and processing of important features from the input data. The architecture culminates in an output layer of two neurons. These output neurons specifically represent the lateral forces at the front and rear axles, respectively. The KAN network, implemented using PyTorch, incorporates learnable activation functions to enhance model adaptability and performance. The network was trained for 500 epochs with a learning rate set at 0.001. For the dataset distribution, 80% was allocated for training purposes, while the remaining 20% was employed for testing. To further refine the model parameters during the later stages of training, the ExponentialLR learning rate scheduler from PyTorch was employed. This scheduler decreases the learning rate by a certain proportion every epoch. The decay factor of the scheduler was set to 0.99. Additionally, to address the dimensional discrepancies between features and labels, we employed the Z-score normalization method. This technique standardizes the data to a normal distribution, achieving a mean of 0 and a standard deviation of 1 for each feature. As depicted in Figure 5, the training curve of the KAN network demonstrated efficient learning, characterized by a steady decrease in MSE (Mean Squared Error) loss and no indications of overfitting. This suggests that the model generalizes well to new data.

**Figure 6:**
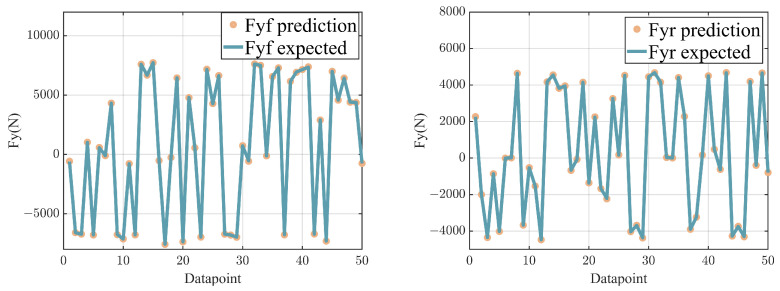
Prediction vs. ground truth.

In the testing phase, we assessed the performance of the KAN network by comparing the estimated values of lateral forces at the front and rear axles with their corresponding actual values. The representative results from this evaluation are illustrated in Figure 6. The experimental results demonstrate that the KAN network has high predictive accuracy on the test dataset, accurately predicting the lateral forces of the front and rear axles of the vehicle.

### 4.2. Double-Lane Change

The double-lane change maneuver is a critical scenario for assessing a vehicle’s ability to safely execute overtaking maneuvers, as illustrated in Figure 4, Case 1. We set the vehicle’s speed at 72 km/h and specified a road surface adhesion coefficient of 0.8. The design parameters for DD-PTC and MPC were identical, with a prediction horizon of $N_{p}=35$, a control horizon of Nc=15, weights for the output variables $Q={\left[\begin{array}{cc} 2000 & 12000 \end{array}\right]}^{T}$, and weights for the control increments R=5000. The weights of the MPC were set to be the same as those of the DD-PTC, and the preview time for LQR was 0.1 s. The lateral force estimation and comparative results of path tracking performance across the three controllers are presented below.

**Figure 7:**
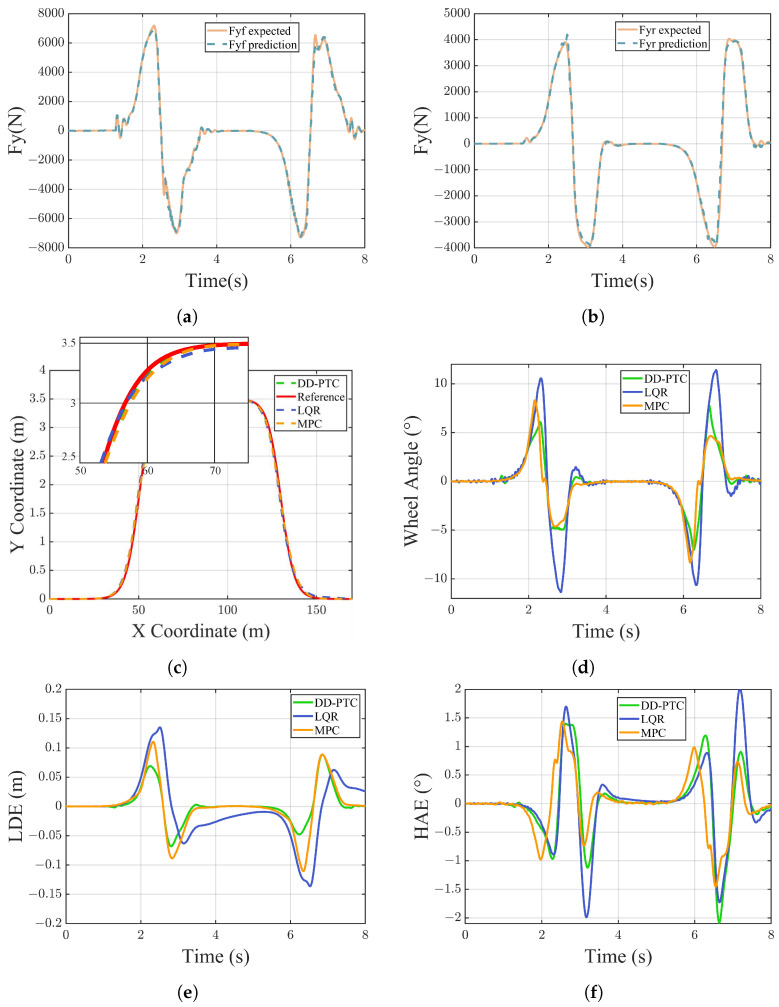
Vehicle simulation results of double-lane change. (**a**) Lateral force of front axle; (**b**) lateral force of rear axle; (**c**) result of path tracking; (**d**) wheel angle; (**e**) lateral distance error; and (**f**) heading angle error.

Figure 7a,b demonstrate that the KAN network exhibited excellent adaptability in estimating lateral forces. The KAN network effectively understood the characteristics of input data and accurately estimated the lateral forces of the front and rear axles of the vehicle. The root mean square errors (RMSEs) of the lateral force estimation for the front and rear axles were 190.1904 N and 136.3055 N, and the maximum estimation errors were $1.093\times 10^{3}\;N$ and 466.854N, respectively. The average absolute errors of the lateral force estimations were 94.5349 N for the front axle and 62.6035 N for the rear axle. Although these average errors were very small, the maximum values were relatively large. This discrepancy may be attributed to the limited data collected under extreme driving conditions, leading to insufficient training of the model. As a result, the network may struggle to accurately estimate lateral forces, so it is necessary to employ more data to thoroughly train the KAN network to enhance its robustness and accuracy under various driving conditions, which will be a focus of future work. Figure 7c demonstrates that all three methods are capable of tracking the reference path. However, in the scenario involving paths with slightly large curvatures, the LQR method exhibited significant lateral errors and slower convergence compared to the other methods. MPC, by predicting future states with its predictive model, converged over more quickly but still exhibited substantial lateral errors due to limitations in its predictive model. In contrast, DD-PTC, which utilizes a more accurate predictive model, demonstrated smaller lateral errors. This distinction underscores the importance of model accuracy in reducing prediction error and enhancing overall system performance. Figure 7d–f and Table 2 provide a detailed comparison of tracking errors for the three controllers. LDE (Lateral Distance Error) and HAE (Heading Angle Error) are key performance metrics. Employing DD-PTC for path tracking significantly improved the performance: the maximum lateral error decreased by 19.83% and 35.02% compared to MPC and LQR, respectively. Additionally, the average lateral error displacement was reduced by 29.56% and 54.51%, respectively. Regarding the heading angle error, the maximum and average values for DD-PTC were $2.0818^{\circ }$ and $0.3564^{\circ }$, respectively, which are roughly equivalent to those recorded for MPC and LQR, indicating that both remained at a comparably low level.

### 4.3. Single-Lane Change

To further validate the robustness of the proposed algorithm, a lane changing simulation experiment was conducted, as illustrated in Figure 4, Case 2. The vehicle speed was fixed at 72 km/h, with a road surface adhesion coefficient of 0.8. The controller parameters were the same as those used for the double-lane change setup. The lateral force estimation and comparative results of path tracking performance across the three controllers are presented below.

**Figure 8:**
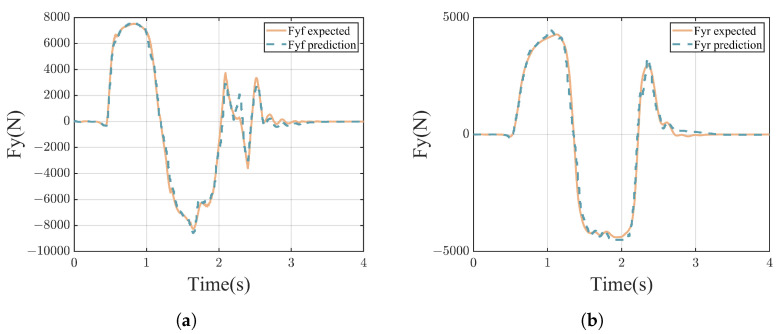

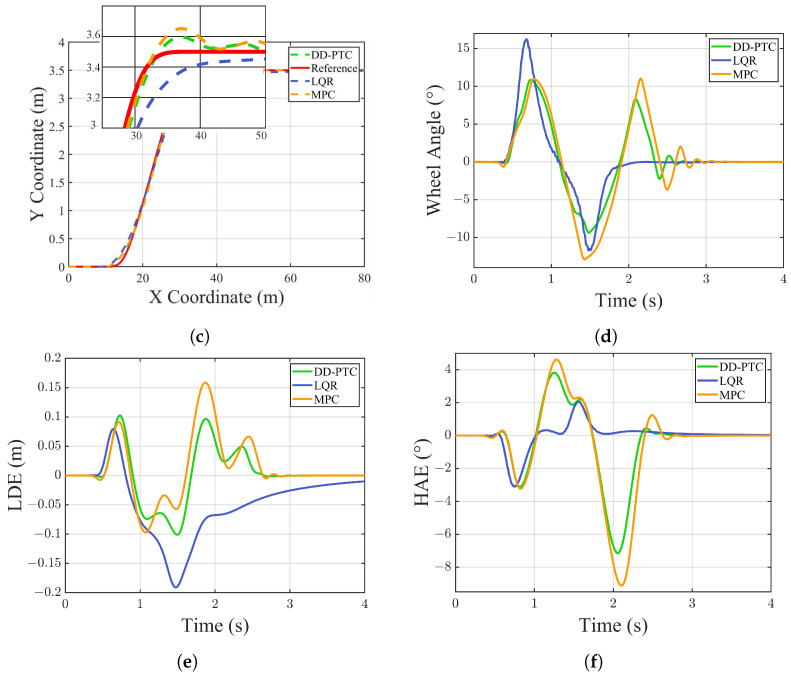
Vehicle simulation results of double-lane change. (**a**) Lateral force of front axle; (**b**) lateral force of rear axle; (**c**) result of path tracking; (**d**) wheel angle; (**e**) lateral distance error; and (**f**) heading angle error.

Figure 8a,b demonstrate that the KAN network generally provided accurate estimates of the lateral forces on the front and rear axles of the vehicle. Most of the estimation results were close to the actual values, with only a few showing some deviations. The RMSE of the lateral force predictions for the front and rear axles were 406.6039 N and 234.9228 N, and the maximum prediction errors were $1.731\times 10^{3}$ N and $1.009\times 10^{3}$ N, respectively. The average absolute errors of the predicted lateral forces for the front and rear axles were 252.2186 N and 145.5211 N, respectively. Notably, in order to reduce the computational load, the KAN network estimated lateral forces without requiring historical data, considering only the current state of the vehicle. Despite this simplification, the network still achieved results that are relatively satisfactory. However, some estimates still exhibited errors, indicating potential for further optimization of the KAN network. Figure 8c–f and Table 3 display the path tracking performance of the three controllers, all demonstrating the ability to track the reference path. In the single-lane change scenario, both MPC and LQR exhibited significant lateral errors. LQR converged more slowly than MPC and DD-PTC, but due to its preview mechanism and slower convergence rate, its HAE was smaller; however, its LDE was significantly greater than those of MPC and DD-PTC. In comparison, DD-PTC performed the best among the three controllers, significantly enhancing path tracking precision, particularly in terms of lateral error. When using DD-PTC for path tracking, the maximum lateral error decreased by 32.64% and 46.30% compared to MPC and LQR, respectively, and the average lateral error decreased by 12.57% and 36.40%. Regarding HAE, the maximum and average values for DD-PTC were $7.1496^{\circ }$ and $1.4074^{\circ }$, respectively, which represents a reduction of 21.60% and 21.92% compared to MPC.

### 4.4. Ablation Experiment

To comprehensively demonstrate the effectiveness of our proposed method, we performed ablation experiments in both single-lane and double-lane change scenarios. Our method was compared with MPC using only cornering stiffness correction, GP-MPC, and standard MPC. The comparison results are presented below.

**Figure 9:**
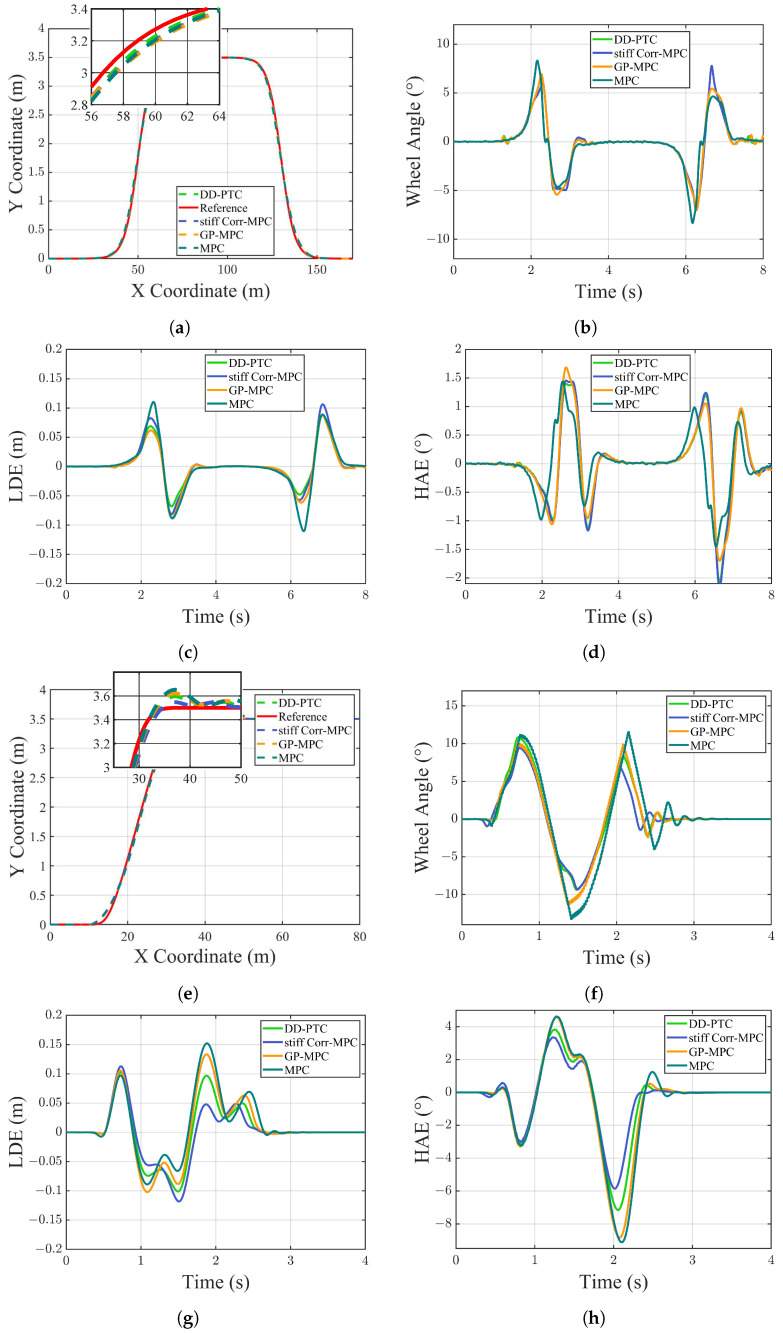
Vehicle simulation results of double-lane change. (**a**) Path tracking results for DLC; (**b**) wheel angle for DLC; (**c**) lateral distance error for DLC; (**d**) heading angle error for DLC; (**e**) path tracking results for SIL; (**f**) wheel angle for SIL; (**g**) lateral distance error for SIL; and (**h**) heading angle error for SIL.

In Figure 9, GP-MPC refers to an MPC approach where only Gaussian Process Compensation was applied to the prediction equation, while Stiff Corr–MPC represents an MPC method with cornering stiffness correction. The results indicate that under both single-lane and double-lane change conditions, the DD-PTC method demonstrated strong performance. Compared to standard MPC, both Stiff Corr–MPC and GP-MPC performed better. The performance of both Stiff Corr–MPC and GP-MPC is presented in Table 4. In the double-lane change scenario, the mean lateral error of DD-PTC was approximately 16.60% lower than that of Stiff Corr–MPC and 9.07% lower than that of GP-MPC, with the maximum error reduced by around 16.67% and 0.65%, respectively. In the single-lane change scenario, although the mean lateral error of DD-PTC was approximately 10.10% higher than that of Stiff Corr–MPC, the maximum error in this scenario was reduced by approximately 13.23% compared to Stiff Corr–MPC. Additionally, the mean lateral error of DD-PTC was 5.58% lower than that of GP-MPC, with the maximum error reduced by 15.52% compared to GP-MPC.

## 5. Discussion and Conclusions

This paper developed and validated data-driven enhancements for the MPC-based path tracking controller, which integrates cornering stiffness corrections and Gaussian Process Regression as residual dynamics to improve both the adaptability of the prediction model and the accuracy of path tracking. Specifically, the strategy utilizes the KAN network to estimate the tire lateral forces for dynamically adjusting cornering stiffness, and combined Gaussian Process Regression to correct discrepancies between the simplified mechanistic model and the actual vehicle state. Our predictive model accurately captures and responds to dynamic changes in the vehicle. Consequently, this enhances the precision of path tracking. In comparative tests conducted on the Simulink-Carsim joint simulation platform, our DD-PTC method demonstrated superior performance in high adhesion double-lane change and single-lane change scenarios, outperforming normal MPC and LQR methods.

Although DD-PTC has demonstrated significant potential, there remains considerable room for optimization. The robustness and precision of the KAN network in estimating lateral forces require further enhancement. Additionally, the computational speed of DD-PTC presents opportunities for optimization. Future research will focus on enhancing the continuous learning capabilities of the KAN network and improving the computational efficiency of DD-PTC. These efforts aim to enable quicker controller responses to changes in complex environments, comprehensively enhancing the controller’s performance and adaptability.

## Figures and Tables

**Table 1:** Vehicle parameters.

Parameter	Value	Parameter	Value
Vehicle mass (kg)	1270	Yaw moment of inertia (kg·m^2^)	1536.7
Wheelbase (m)	2.910	Wheel track (mm)	1.675 (front, rear)
Distance from front axle to center of mass (m)	1.015	Distance from rear axle to center of mass (m)	1.895
Centroid height (m)	0.54	Rolling radius (m)	0.325
Front axle cornering stiffness (N/rad)	60,000	Rear axle cornering stiffness (N/rad)	40,000

**Table 2:** Double-lane change test.

	LDE (m)	HAE (°)
	Max/Avg.	Max/Avg.
DD-PTC	0.0885/0.0162	2.0818/0.3564
LQR	0.1362/0.0357	2.0139/0.4071
MPC	0.1104/0.0230	1.4532/0.3080

**Table 3:** Single-lane change test.

	LDE (m)	HAE (°)
	Max/Avg.	Max/Avg.
DD-PTC	0.1025/0.0331	7.1496/1.4074
LQR	0.1908/0.0520	3.1016/0.3942
MPC	0.1521/0.0378	9.1182/1.8024

**Table 4:** Ablation study results of path tracking performance in single-lane and double-lane change scenarios.

	Test Scenarios	LDE (m)	HAE (°)
		Max/Avg.	Max/Avg.
Stiff Corr–MPC	Double-lane	0.1062/0.0194	2.1643/0.3705
	Single-lane	0.1181/0.0297	5.8537/1.1589
GP-MPC	Double-lane	0.0891/0.0178	1.6996/0.3610
	Single-lane	0.1213/0.0350	8.8269/1.7213

## Data Availability

No new data were created or analyzed in this study. Data sharing is not applicable to this article.
